# Optical Thermography Infrastructure to Assess Thermal Distribution in Critically Ill Children

**DOI:** 10.1109/OJEMB.2021.3136403

**Published:** 2021-12-17

**Authors:** Monisha Shcherbakova, Rita Noumeir, Michael Levy, Armelle Bridier, Victor Lestrade, Philippe Jouvet

**Affiliations:** Ecole de Technologie Superieure 514734 Montreal QC H3C1K3 Canada; CHU Sainte Justine Mother and Child Hospital of Montreal 568063 Montreal QC H3T1C5 Canada; Ecole de Technologie Superieure 514734 Montreal QC H3T1C3 Canada; Pediatric Intensive Care Unit of CHU Sainte Justine Mother and Child Hospital of Montreal 568063 Montreal QC H3T1C5 Canada

**Keywords:** Critical care, hemodynamic stress, infrared thermography, IR cameras, thermal gradients

## Abstract

The temperature distribution at the skin surface could be a useful tool to monitor changes in cardiac output. *Goal:* The aim of this study was to explore infrared thermography as a method to analyze temperature profiles of critically ill children. *Methods:* Patients admitted to the pediatric intensive care unit (PICU) were included in this study. An infrared sensor was used to take images in clinical conditions. The infrared core and limb temperatures (θ_c_ & θ_l_) were extracted, as well as temperatures along a line drawn between these two regions. *Results:* The median [interquartile range] θ_c_ extracted from the images was 33.88°C [32.74-34.19] and the median θ_l_ was 30.21°C [28.89-33.13]. There was a good correlation between the θ_c_ and the clinical axillary temperature (rho = 0.39, p-value = 0.016). There was also a good correlation between the θ_c_ and θ_l_ (rho = 0.66, p-value = 1.2 e^−05^). *Conclusion:* Thermography was found to be effective to estimate the body temperature. Correlation with specific clinical conditions needs further study.

## I. Introduction

Humans have the ability to regulate their own body temperature, and keep it at a stable level between 36.5 °C and 37.5 °C on average. Similarly, the temperature of the surface of the skin remains stable in the range of 33°C to 36 °C under normal conditions. [1], [2] The temperature of the skin and various parts of the body is a direct result of the blood flow, and hence the temperature distribution at the surface of the skin can give us an insight into the changes in vascularization in the body. [3] This can allow us to understand whether the blood flow is centralized to the vital organs and directed away from the superficial organs and limbs. Studies have shown that patients with fever and shock show abnormal temperature gradient along the limbs. [4], [5] One study also found that the temperature distribution in children follows a similar pattern to adults, but with less variation. [6] This type of hemodynamic analysis of the human body based on temperature is a new and diverse field and is especially novel when applied to children in the pediatric intensive care unit (PICU).

Infrared thermography (IRT) is a non-invasive method of temperature measurement. All objects that have a temperature above absolute 0 K (−273 °C) emit infrared radiation. These infrared rays when detected by a sensor can be used to measure the objects temperature. Over the last decade, the prevalence and use of infrared sensors for body temperature measurement has surged. The efficacy of these sensors in the clinical setting is yet to be studied and validated for widespread use, especially in the case of critical care. The International Standards Organization (ISO) published a protocol for the use of IR cameras for temperature estimation and recommended that the inner canthus region of the eye is the only site on the face that is suitable for non-contact temperature measurement [7]. Hence, it is recommended for any studies that use IR cameras for core temperature detection to only use the inner canthus region of the eye to get the most accurate possible measurements.

The non-contact nature of IR screening is its biggest appeal in the field of pediatrics, as other forms of temperature measurement such as rectal thermometers are more invasive and cause discomfort to the patients without giving an insight into the temperature distribution throughout the body [8]. A study analyzing 483 examinations performed on 285 pediatric patients found that IRT was a useful tool to follow up and supplement the diagnosis and detection of various ailments such as vascular malformations, burns, and thrombosis [9]. Even though the use of IRT in pediatrics is still a new field due to a limited number of relevant studies, it seems to be more accurate in skin temperature estimation in children as compared to adult subjects [10].

Therefore, the aim of this study was to validate IRT as a reliable estimate of core body temperature in critically ill infants and analyze the clinical value of temperature evolution across the body assessed by IRT.

## II. Materials and Methods

### A. Inclusion Criteria

Patients less than 18 years of age, admitted to the PICU of Centre Hospitalier Universitaire Sainte-Justine (CHUSJ) between January 1st, 2021 and May 30th, 2021 with vital distress (respiratory, hemodynamic or neurological), were prospectively included in this study, after obtaining written informed consent.

### B. Choice of Cameras

Our setup consisted of two cameras – the Kinect Azure and the FLIR Lepton 3.5 LWIR (Long Wave InfraRed) thermal infrared sensor. The sensor had a resolution of 160 x 120 pixels with a thermal sensitivity of < 50 mK. The Azure has several operating modes for its RGB camera, the highest resolution being 4096 x 3072 pixels.

The camera was embedded in the FLIR Breakout Board V2, and the board was connected to a Raspberry Pi 3B + that was used to interface with the camera and acquire the images.

The acquisition of RGB videos and images by the Kinect allowed us to get a clear visible light image of the patient to identify any obstructions or artefacts (such as medical equipment) on the patient's body. In addition, the camera was used to extract 3D point cloud videos of the patient, to be used in other projects that assess the respiratory profile conducted at the same institution [11], [12].

### C. Calibration of Cameras

To automate this procedure of temperature estimation, the RGB images from the Kinect were superimposed with the IR image from the Lepton. The procedure of calibrating the two cameras involved taking photos of a calibration marker, whose dimensions are known. The calibration marker used was a checkerboard pattern, provided by MATLAB [13]. Images of this marker were taken from both cameras, and the checkerboard was detected in each image using object detection. A special marker made of aluminum squares instead of ink was used for the IR sensor, so that the squares show up clearly in the IR image. As aluminum has a low IR emissivity, it shows up dark in the image, while the rest of the marker remains white.

### D. Image Registration

**Fig. 1.**
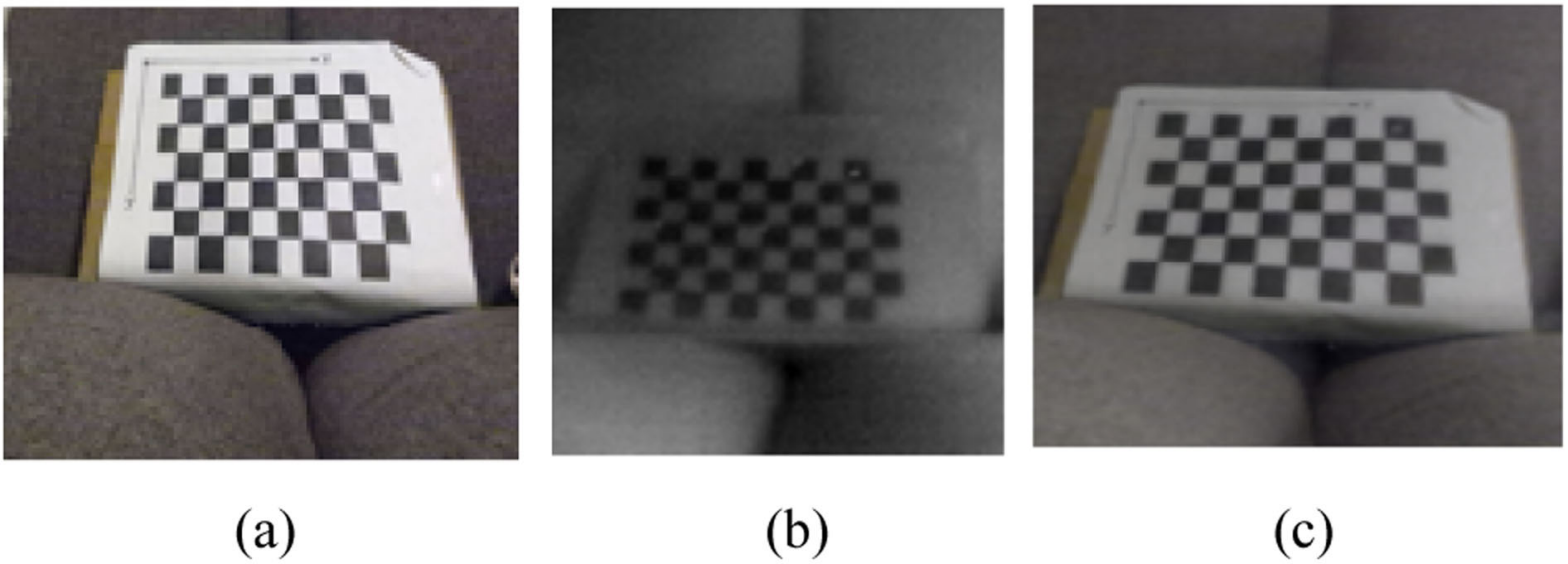
In subfigure (a) is a sample image taken with the Kinect Azure. Subfigure (b) shows a sample image taken with the Lepton IR sensor. The subfigure (c) is the result of superposition of these two images after registration.

The process of calibrating the cameras together in MATLAB provides a transformation matrix, that defines how one image must be warped to be superimposed accurately over the other camera image. The checkerboard is detected in the image using the Camera Calibrator App from MATLAB. The points are used to estimate a transformation matrix that is used to warp the IR and RGB images over each other. The result of this superposition is shown in Fig. 1.

### E. Temperature Measurement and Extraction

The FLIR Lepton 3.5 sensor has a radiometry feature, which essentially means that the temperature values for each pixel can be extracted from the pixel values of the image. When the TLinear mode is enabled, the image pixel values are converted from representing the scene flux in 14-bit digital counts to representing temperature values in Kelvin. If a pixel value is 30000, that means the pixel is at 300.00 K or 26.85 °C. [14]

In the future, when this process will be automated, one will be able to extract the pixel values, and in turn, the temperature values from a specific region of the image, defined by the skeleton of the patient that will be obtained from the Kinect image. Therefore, the estimation of the core and limb temperature will be possible with little to no human intervention.

### F. Assembly of All Parts in the Hospital

The set up used at the hospital consisted of a PC and the combined camera (Kinect Azure and IR sensor) embedded in a support. The whole setup of the equipment in the hospital was done on a movable trolley. The support with the Azure camera and the Lepton were connected to a pole that can be fixed onto the edge of the pediatric intensive care unit (PICU) beds, allowing the cameras to be pointed towards the patient at an appropriate distance without interfering with the work of caregivers. The entire setup was reviewed by the IT department at the CHUSJ and approved for use in the PICU unit.

### G. Ethics and Legal Compliance

This study was observational. The study protocol and the experimental equipment setup was approved by the Research Ethics Board (2016 project number 1242) before being used in the pediatric intensive care unit (PICU).

### H. Performing Acquisitions With Patients

The equipment set up was brought into the ICU room and the camera placed at the edge of the bed. The chest of the patient was exposed, and any clothing or blankets were removed. The hands and feet were exposed so that temperatures of the extremities can be extracted. During the acquisition of 3D point clouds and RGB videos, in order to avoid disturbance in the field of view obscuring the view of the patient, parents and caregivers were asked to vacate the area for the duration of the acquisition. The required images and videos were recorded, and the entire procedure took about 10 minutes.

### I. Extraction of Temperatures From IR Images

**Fig. 2.**
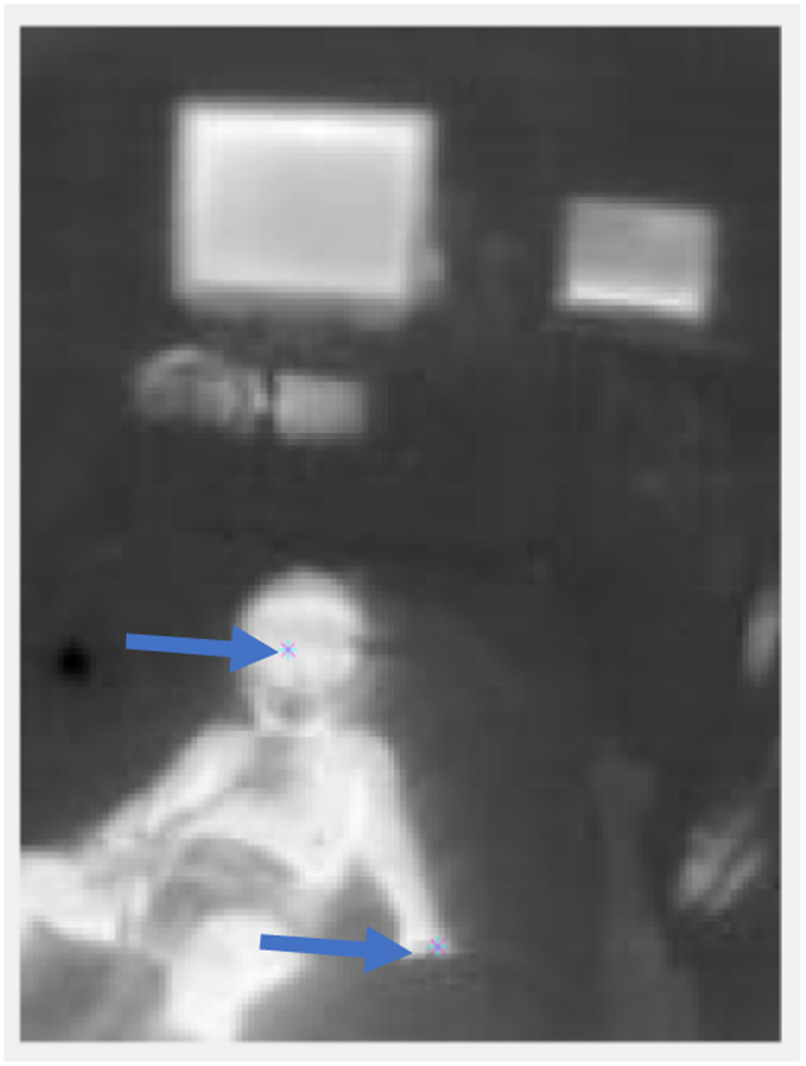
An IR image taken of a patient as part of the study. Two points marked with a small cross have been selected in the image, one on the face and one on the hand of the patient to extract temperatures of corresponding locations.

To determine the core temperature (estimated as temperature of the inner canthus) and the temperature of the extremities (namely either the toes or fingers), the pixel values of the corresponding regions were extracted manually, using a software developed in MATLAB. In some images, the eyes of the patient were not visible or obstructed, in this case the thorax was considered for core temperature. Another point from either the toes or fingers was taken, whichever was more clearly visible. Next the difference between these two values was calculated as the gradient. Fig. 2 shows the points at which the temperatures were extracted for a sample patient.

### J. Use of RGBD Data to Identify Obstructions on Patient Body

Each acquisition per patient comprised of a few IR images and an RGB video. IR images only gave us a thermal heat map of the scene, while RGB images gave us the ability to recognize and identify objects, which can be of use when identifying the exact position of the patient's body, and the presence of any medical equipment that may cause obstructions in temperature extraction. If the temperature extraction procedure is performed while only referencing the IR images, because the artefacts are not visible, the temperature points selected may not be accurate.

### K. Analysis of Temperature Along the Line Joining the Core to the Extremities

A useful insight into the thermal heat map of the patient would be to explore how the temperature varies along a line that joins the core (e.g., the inner canthus) to the end of the limbs, say to the fingertips. This would give us a look into how the body temperature changes as we go from the core of the body towards the extremities. These temperature changes can reflect blood flow distribution and gives clinical information on the patient's hemodynamic status.

The analysis was carried out in MATLAB. A line was drawn over the IR image to connect the core to the end of either the hand or the feet. A sample of points was extracted from the line, and the corresponding pixel values were converted to temperature. These values were then plotted on a graph to give a visual representation of how the temperature varies along the body.

### L. Clinical Data Recorded for Analysis

The core temperature taken by nurses at the time that is closest to the time of acquisition was recorded. This temperature was either a rectal temperature or an axillary temperature. The axillary temperature taken by the nurse is incremented by 0.5 °C when recorded in the electronic medical records (EMR), to make it equivalent to the rectal core temperature measurement [15].

The patients were labeled by two physicians according to their clinical state at the time of the acquisition, using data from their EMR. The clinical states analyzed were the presence of any clinical sign of decrease or increase in cardiac output (diagnosis of shock, tachycardia, vasoconstrictor/vasodilator drugs infused, hypotension, diagnosis of cardiac failure, cardiac surgery).

The PICU rooms have a constant ambient temperature of 21°C – 22 °C.

### M. Statistical Analysis

Data were presented as median and interquartile ranges. Spearman correlation studies were performed using the gradients and ratios based on clinical states, using R software [16].

## III. Results

In total, 42 patients were included. Thirty-six patients out of 42 patients had clear and usable IR images. Six of the patients had to be excluded due to distortions in the images (n = 3), incorrect temperature calibration (n = 1), or because their limbs were not visible (n = 2).

Their median age was 8 months old, and the median weight was 7.4 Kg. Of the 36 subjects, 16 were female. The detailed description of the patient sample is shown in Table I.

**TABLE I:** Study Population

		**N=36**	
**Age (median month)[interquartile range]**		8 [1-64.5]	
**Weight (median Kgs)[interquartile range]**		7.4 [4.5-24.6]	
**Female sex**		16 (44%)	
**Reason for admission**			
Respiratory distress		12 (33.34%)
Post-cardiac surgery		10 (27.77%)
Other		14 (38.89%)
**Medical history**			
Chronic underlying diseases		33 (91.67%)
None		3 (8.33%)
**Shock state during recording** (Cardiogenic shock treated with milrinone)		2 (6%)	
**Temperature (median C) [interquartile range]**		36.8 [36.3-37]	
**Site**			
axilary		34 (94%)	
central		2 (6%)	
**Hemodynamic support (HS)**		10 (28%)	
**Type of HS**			
Vasopressors		3/10 (30%)
Vasodilators		7/10 (70%)
**Respiratory support (RS)**		18 (50%)	
**Type of RS**			
Ventilatory support with endotracheal tube		6/18 (33.34%)
Ventilatory support with facial or nasal mask		10/18 (55.55%)
Other ventilatory support		2/18 (11.11%)
	

*Note:* Table showing the physiological and clinical markers of the subject sample.

The reason for admission for these patients were diverse, with 12 admitted for respiratory distress, 10 after a cardiac surgery, while the remaining varied from cardiac failure, arrythmia, pulmonary hypertension, and neurosurgery, among others. Most of them had an underlying chronic disease such as respiratory failure, premature birth, and kidney failure.

Of the 36 patients, 10 were on continuous perfusion of vasoactive drugs. Eighteen of the 36 patients were on respiratory support, and the types of support are described in Table I.

### A. IR Temperature Accuracy and Temperature Gradient Analysis

The median central temperature extracted from the IR images of the subjects was 33.88°C [32.74-34.19 °C], and the median temperature of the extremities was 30.21°C [28.89-33.13 °C]. There was a good correlation between the central temperature extracted via thermography and the clinical axillary temperature, with a Spearman's correlation factor of 0.40 (p-value of 0.017). There was also a very good correlation between the central and extremities temperature extracted via thermography with a Spearman's correlation factor of 0.66 (p-value = 1.2 e-05). This shows that there is consistency between temperatures extracted from different parts of the body for the same patient (the scatter plots for both analyses are included in the Supplementary Materials section).

The gradient between the central and extremities temperature of the patients was calculated by taking the difference between the two temperature values. The median gradient for the subject sample was 3.19 °C [1.06-4.64] and the maximum gradient was 5.96 °C.

**Fig. 3.**
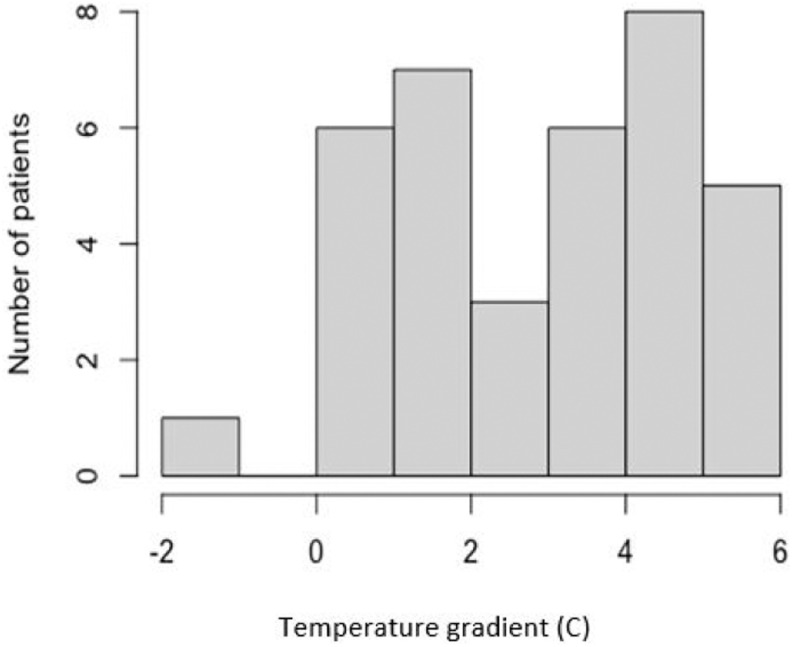
Histogram of temperature gradient vs number of subjects. The x axis denotes the temperature gradient in degree C (bins of 2 C), and the y axis is the number of patients that had gradients in that bin.

Fig. 3 depicts the distribution of this gradient for the entire subject sample. As can be seen from the graph, the gradient values were varied, ranging from −1.14 °C to + 5.96 °C.

**Fig. 4.**
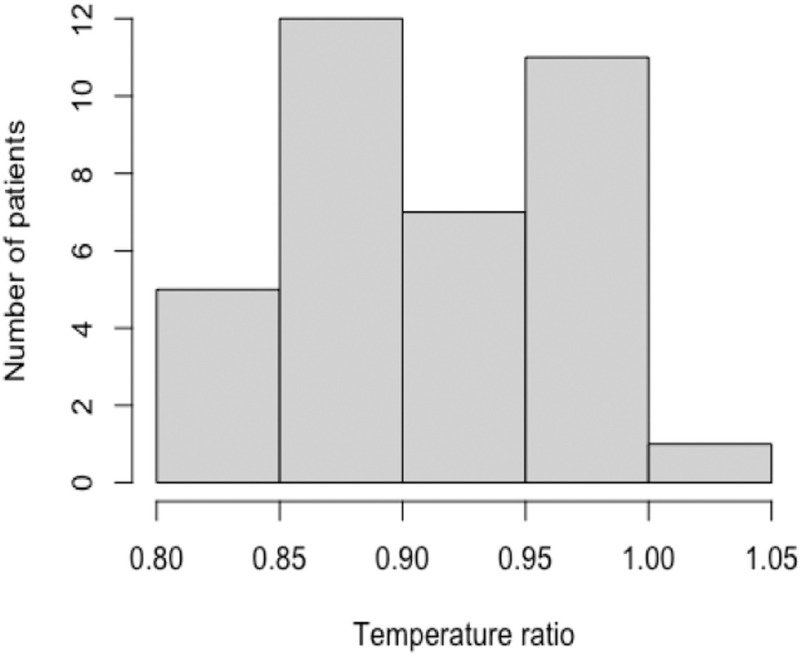
Histogram of temperature ratio vs number of subjects. The x axis is the temperature ratio binned on 0.05, and the y axis is the number of patients with ratios falling in that bin.

Most of the patients (except one) tended towards positive gradients, meaning that the core was warmer than the extremities, which was the expected result. The median temperature ratio calculated for the subject sample was 0.90 [0.86-0.96], and the maximum ratio was 1.03. Fig. 4 shows the histogram distribution of the ratios for the entire sample. The ratio is calculated by dividing the temperature of the extremities by the temperature of the core, both extracted from the IR image of the patient. The ratios ranged from 0.8 to 1, with only one patient displaying a ratio > 1. The same patient demonstrated a negative gradient, meaning that the subject's extremities were warmer than the core. The patient's temperature gradient was 1.14 °C and the temperature ratio was 1.03. This subject was receiving milrinone, a vasodilator drug. The gradient for the patients with vasodilators (n = 7) was 3.14 ± 2.49 and for the patients with vasoconstrictors (n = 3) 3.37 ± 1.94. When data acquisition was done, hemodynamic stability was almost restored in most patients, using vasoactive drugs. This may explain why the gradients were not different between the groups.

### B. Temperature Gradient Correlation With Clinical Status

There was no significant correlation between neither thermal gradient and clinical status nor temperature ratio and clinical status. The results for the correlation analysis are shown in Table II. Based on p-values, there are no subgroups that show significant correlation.

**TABLE II:** Impact of Clinical Factors on Temperature Gradient Between Core and Extremities Recorded by Thermography

**Group 1 vs Group 2**	**Median Group 1 [Q1-Q3]**	**Median Group 2 [Q1-Q3]**	**p value**
**Admitted Post cardiac surgery vs No**	2.28 [1.20-4.39]	3.41 [1.07-4.68]	0.7678
**Admitted For cardiac failure vs No**	3.34 [1.69-4.34]	3.19 [1.06-4.64]	1
**History of Congenital heart disease vs No**	2.66 [0.94-4.17]	3.81 [1.11-4.76]	0.3736
**History Neurologic disease vs No**	4.31 [3.69-4.78]	2.63 [1.06-4.48]	0.1782
**Shock vs No**	3.54 [2.72-4.37]	3.19 [1.06-4.58]	0.5778
**Hypotension vs No**	4.94 [3.29-5.07]	3.04 [1.06-4.48]	0.2661
**Tachycardia vs No**	3.97 [3.83-4.58]	2.65 [1.06-4.56]	0.3274
**Hemodynamic support vs No**	3.42 [1.92-4.57]	3.00 [1.03-4.58]	0.6388
**Vasodilator support (milrinone) vs No**	4.05 [3.81-5.19]	2.66 [1.06-4.55]	0.3476

*Note:* The above table shows the results of the correlation analysis done based on temperature gradient. The first and second columns of mean and SD values correspond to the respective subject groups. In this analysis, the samples were not segregated by gender. A separate gender based analysis was performed and gave no significant results.

### C. Temperature Analysis Along the Line Joining the Core to the Extremities

**Fig. 5.**
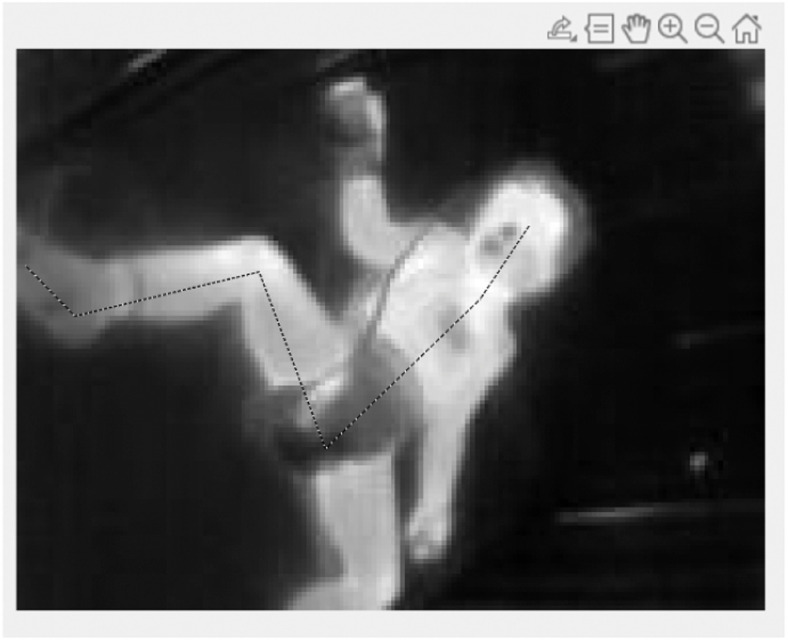
IR image taken of another patient as part of the study. A line is drawn starting from the inner canthus region of the eye, down to the chest and along the leg to the toes.

**Fig. 6.**
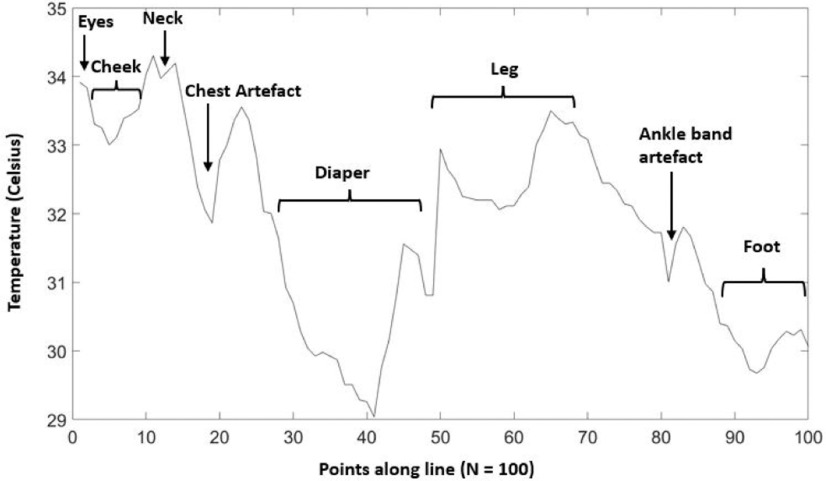
Resulting graph for the line drawn in the above image.

Fig. 5 depicts the line that was drawn on the IR image of a patient, from the eyes down through the thorax to the toes. This line was used to create the resulting graph depicted in Fig. 6. The temperatures of each pixel along the line were extracted and their values plotted. The graph goes from a high peak that represents the temperature of the eyes, as the line moves over the cheek, which is visibly cooler in the IR image, we can see a drop in the graph line. As the line moves over the neck the temperature increases again, but as the line moves over the chest artefact, which was a bandage, there is a sharp downward drop in the graph. Similarly, there is a large trough that corresponds to the temperature as the line moves over the diaper region of the patient.

## IV. Discussion

Despite the absence of significant results from the hemodynamic assessment using IRT in our cohort study, we believe that IRT can be a useful tool in evaluating the thermal profile of critically ill patients, especially to monitor the impact of the vasoactive support on this gradient with time (not assessed due to study design). The graph drawn using the temperatures extracted along the line in Fig. 5 and 6 demonstrates an opportunity to evaluate the continuous gradient present throughout the limbs. For example, the section of the graph line that corresponds to the leg can be used to calculate the slope of the temperature gradient from the thorax to the toes. This slope can then be correlated with clinical vital sign markers such as oxygen extraction ratio to study if it is correlated with this marker of cardiac output. A threshold can be used to eliminate temperature values outside a certain range, and this could be used to automatically detect artefacts present on the patient's body.

The statistical correlation analysis done demonstrates potential to quantify the gradient that exists between different parts of the body of critically ill children. The temperatures recorded from the IR images are in line with the literature on the temperature of skin in children, but previous work focused mostly on healthy subjects [6]. Although the patients recruited here were from a diverse clinical background, they were all stabilized at the time of acquisition. In addition, the sample was unbalanced, for example only 10 patients were on hemodynamic support, while the rest 26 were not. In our sample, the one patient with higher temperature in the extremities was a patient under vasodilating medication. However, we could not draw any conclusion due to our small sample size and the absence of evolution of temperatures over time for a given patient.

One of the main limitations experienced was the low resolution of the IR sensor. For the checkerboard to be clearly visible in the IR image, it must be placed close to the sensor, roughly 1 meter away from the camera. If the checkerboard is placed far away, it becomes difficult for the checkerboard detection algorithm to detect the squares. Registering the image views of cameras that have an extreme difference in resolution is challenging. This limitation will have to be addressed in the future, either by using a different marker or upgrading the IR sensor to one with a higher resolution.

## V. Conclusion

In conclusion, the study explored several pathways for which IRT could be useful for temperature screening of critically ill children. More work needs to be done to improve the registration algorithm and perform analyses on a more diverse subject sample with patients demonstrating vital distress.

## Supplementary Materials

Supplementary materials
