# Supplementary material for: Optical Thermography Infrastructure to Assess Thermal Distribution in Critically Ill Children
Source: IEEE Open J Eng Med Biol. 2021 Dec 17;3:1–6. doi: 10.1109/OJEMB.2021.3136403 (PMC8975240; doi:10.1109/OJEMB.2021.3136403)
Supplement: Supplementary materials [file supp1-3136403.pdf]

IEEE Open Journal of
Engineering in Medicine and Biology

Optical thermography infrastructure to assess thermal distribution in critically ill children

Monisha Shcherbakova, Rita Noumeir Ph.D., Michael Levy M.D., Ph.D., Armelle Bridier M.D., Victor Lestrade M.D., Philippe Jouvet M.D., Ph.D.

SUPPLEMENTARY MATERIAL

The below supplementary material includes some figures that were not included in the main body of the article.

The following graphs depict some of the correlation analyses performed as part of this work. The first graph (Figure 7) depicts the scatter plot of the clinically measured axillary temperature versus the core temperature extracted via thermography. The p value is 0.017 and the Spearman's correlation factor of 0.40.

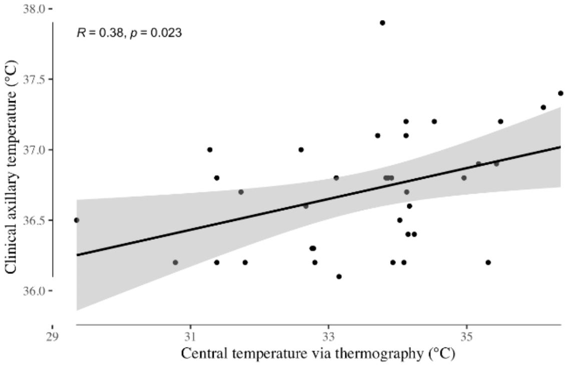

Figure 7: Scatter plot with regression line for the correlation between the clinical axillary temperature and the extremities temperature extracted vis thermography.

The next graph (Figure 8) depicts the correlation between the temperature extracted from the extremities of the patient, versus the temperature extracted from their core, via thermography. There is a good strong correlation between the two values, which shows that the method has consistency within the same patient (Spearman's correlation coefficient of 0.66, and a p value of 1.2 e-05).

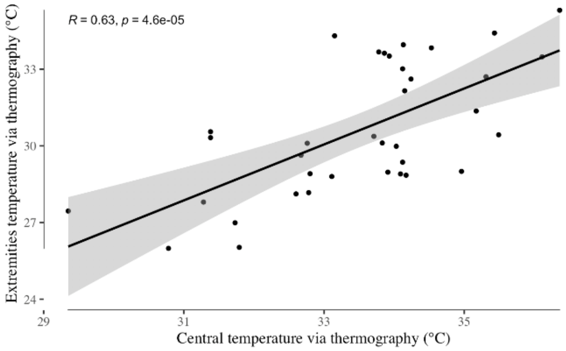

Figure 8: Scatter plot with regression line for the correlation between the clinical axillary temperature and the core temperature extracted vis thermography.
